# Surface Functionalization of Silica Nanoparticles: Strategies to Optimize the Immune-Activating Profile of Carrier Platforms

**DOI:** 10.3390/pharmaceutics14051103

**Published:** 2022-05-21

**Authors:** Benjamin Punz, Litty Johnson, Mark Geppert, Hieu-Hoa Dang, Jutta Horejs-Hoeck, Albert Duschl, Martin Himly

**Affiliations:** Department of Biosciences and Medical Biology, University of Salzburg, 5020 Salzburg, Austria; benjamin.punz@plus.ac.at (B.P.); litty.johnson@plus.ac.at (L.J.); mark.geppert@plus.ac.at (M.G.); hieu-hoa.dang@plus.ac.at (H.-H.D.); jutta.horejs-hoeck@plus.ac.at (J.H.-H.); albert.duschl@plus.ac.at (A.D.)

**Keywords:** NH_2_, COOH, APCs, moDCs, uptake, maturation

## Abstract

Silica nanoparticles (SiNPs) are generally regarded as safe and may represent an attractive carrier platform for nanomedical applications when loaded with biopharmaceuticals. Surface functionalization by different chemistries may help to optimize protein loading and may further impact uptake into the targeted tissues or cells, however, it may also alter the immunologic profile of the carrier system. In order to circumvent side effects, novel carrier candidates need to be tested thoroughly, early in their development stage within the pharmaceutical innovation pipeline, for their potential to activate or modify the immune response. Previous studies have identified surface functionalization by different chemistries as providing a plethora of modifications for optimizing efficacy of biopharmaceutical (nano)carrier platforms while maintaining an acceptable safety profile. In this study, we synthesized SiNPs and chemically functionalized them to obtain different surface characteristics to allow their application as a carrier system for allergen-specific immunotherapy. In the present study, crude natural allergen extracts are used in combination with alum instead of well-defined active pharmaceutical ingredients (APIs), such as recombinant allergen, loaded onto (nano)carrier systems with immunologically inert and stable properties in suspension. This study was motivated by the hypothesis that comparing different charge states could allow tailoring of the binding capacity of the particulate carrier system, and hence the optimization of biopharmaceutical uptake while maintaining an acceptable safety profile, which was investigated by determining the maturation of human antigen-presenting cells (APCs). The functionalized nanoparticles were characterized for primary and hydrodynamic size, polydispersity index, zeta potential, endotoxin contamination. As potential candidates for allergen-specific immunotherapy, the differently functionalized SiNPs were non-covalently coupled with a highly purified, endotoxin-free recombinant preparation of the major birch pollen allergen Bet v 1 that functioned for further immunological testing. Binding efficiencies of allergen to SiNPs was controlled to determine uptake of API. For efficacy and safety assessment, we employed human monocyte-derived dendritic cells as model for APCs to detect possible differences in the particles’ APC maturation potential. Functionalization of SiNP did not affect the viability of APCs, however, the amount of API physisorbed onto the nanocarrier system, which induced enhanced uptake, mainly by macropinocytosis. We found slight differences in the maturation state of APCs for the differently functionalized SiNP–API conjugates qualifying surface functionalization as an effective instrument for optimizing the immune response towards SiNPs. This study further suggests that surface-functionalized SiNPs could be a suitable, immunologically inert vehicle for the efficient delivery of biopharmaceutical products, as evidenced here for allergen-specific immunotherapy.

## 1. Introduction

The past years have witnessed a boost in the importance and growth of novel biopharmaceuticals which include peptides, proteins and antibodies [1]. When we consider the USFDA approvals from the year 2017 to 2019, more than 30% of the approved drugs were biologics (2017: 12 of 34; 2018: 17 of 42; 2019: 10 of 48) [2]. Compared to the traditional small drug molecules, biologics provide high target specificity and pharmacokinetics resulting in minimal off-target effects, making them more favorable [3,4]. Even though they exhibit increased effectiveness for a wide range of diseases including cancer and metabolic disorders, they face major challenges. One of the biggest challenges is the delivery of biologics to achieve their maximum therapeutic potential. This is attributed to their structural complexity, decreased stability, distribution, and permeability across biological barriers [5]. Nanoparticulate systems constitute an effective strategy to improve the delivery of biologics. The biologics can be either encapsulated inside the nanoparticles (NPs) or loaded onto their surface by covalent conjugation or by physical adsorption [6].

Silica NPs (SiNP) are an attractive platform especially for protein delivery due to their excellent biocompatibility, potential for surface modification, safety, tunability, and stability. They have been studied previously for intra- and extracellular protein delivery and enzyme mobilization [7,8,9]. The functionalization of SiNPs offers diverse prospects, especially in the field of protein delivery. Hollow-type mesoporous SiNPs with surface amino functionalization were used for the delivery of bovine viral diarrhea virus protein, and show promising potential for the development of nanoparticle-based recombinant subunit vaccines [10]. Wolley et al. successfully demonstrated the conjugation of platelet activation-specific antibodies to polyamido-amine dendrimer-functionalized SiNPs, facilitating the development of a diagnostic tool in cardiovascular disease [11]. Furthermore, carboxylic acid functionalization of SiNPs has been reported to improve protein-loading efficacy and sustained release, and to improve the particles’ thermal stability and adaptability [12,13]. However, all these benefits remain of limited value if the functionalized nanomaterial exerts an enhanced or altered immunological effect, specifically when directed against the cargo (adaptive immunity). Immunological inertness is thus, for many applications, desired when considering functionalized nanoparticles for drug delivery. Ideally, they should remain undetectable by the innate immune system, display a low propensity to activate the adaptive immune system, and serve the purpose of delivering their cargo effectively to the target site. In this regard, surface functionalization of particulate systems should be scrutinized, as the functional groups or molecules associated with the surface of NPs can cause immunomodulation or induce an unexpected immune recognition resulting in an unwanted response. For example, polyethylene glycol (PEG) can induce an anti-PEG immune response and immunological memory, thereby resulting in reduced clinical efficacy and increased adverse effects [14]. Furthermore, even if the free molecule in solution exhibits weak immunogenicity, it can become immunogenic when associated with a carrier [15,16]. For instance, while PEG by itself is considered as immunologically inert and safe, PEGylation of nanoparticles results in the induction of anti-PEG antibodies, leading to the enhanced clearance of NPs from the blood and hypersensitivity reactions in patients [16]. Moreover, the physicochemical properties of nanomaterials, including their coatings, have been reported to determine the outcome of an immune response [17]. Thus, it is essential to elucidate the immune effects of functionalized nanoparticles at an early stage during development of nanobiopharmaceutics.

Dendritic cells are the most potent antigen-presenting cells (APCs) that can prompt the initiation of a primary immune response. Hence, they are highly relevant cells to investigate the immune effects of functionalized nanomaterials. Several reports have revealed the influence of the physicochemical properties of nanoparticles on the molecular mechanisms leading to an immune response. This includes the recognition by immune cells, efficiency of uptake, APC maturation, antigen processing, presentation, and T cell differentiation [18,19,20]. For example, nanoparticles with an optimum size of 50 nm were found to be taken up more effectively compared smaller or larger particles [21].

In this study, we compared the immune effects of aminopropyltriethoxysilane- (SiNP_A-conferring amino (NH_2_) group) and Meldrum’s acid- (SiNP_M-conferring a carboxy (COOH) group) functionalized silica particles with uncoated, i.e., SiNPs, silica NPs in APCs. SiNP_M was used as an alternative to previously investigated undecanoic acid-functionalized particles due to increased stability in suspension and, thus better performance in immunological assays involving APCs. Dendritic cells derived from human peripheral blood monocytes, termed monocyte-derived dendritic cells (moDCs) were used as APCs to study immune effects. We have previously observed alterations in the antigen processing patterns when Bet v 1 was conjugated to SiNPs due to the structural alteration of the protein at the nanomaterial interface [22]. Thus, the immune effects of the particles with and without conjugation to Bet v 1 (the model protein) were also explored here. Bet v 1, the major birch pollen allergen is a well-characterized, well-studied protein; its properties as an allergen facilitate specific immunological assessments in follow-up studies to determine its suitability as a candidate for allergen-specific immunotherapy. Here, we investigated the two major molecular mechanisms contributing to the initiation or modulation of an immune response, including the uptake of antigen and the maturation of the APCs.

## 2. Materials and Methods

### 2.1. Synthesis and Functionalization of SiNPs

#### 2.1.1. Synthesis of SiNPs

SiNPs used in this study were synthesized by the Stöber method as previously described by Liberman et al. [23]. Briefly, 200 mL ethanol (96%) and 36 mL deionized water were heated to 75 °C in a two-neck round-bottom flask attached to a reflux condenser. To this mixture, 10 mL of 25% aqueous ammonia was added and the system was equilibrated for 15 min. This was followed by the addition of 15 mL tetraethylorthosilicate (TEOS), and the reaction was vigorously stirred for 2 h at 500 rpm. The obtained silica dispersion was centrifuged for 1 h at 6000× *g* at 4 °C. The pellet was then washed three times with deionized water by centrifugation at 6000× *g* for 30 min. Then, the washed particles were suspended in ethanol (96%) to circumvent extensive particle agglomeration, and dried at 80 °C for 12 h.

#### 2.1.2. Functionalization of SiNP with Amino (NH_2_) Groups

In order to attach functional amino groups to the surface of the SiNP, a slightly modified version of the protocol from Avella et al. [24] was followed. In the beginning of this procedure, 200 mg 3-aminopropyltrethoxysilane (APTES) was dissolved in 20 mL of water and kept at room temperature for 2 h under continuous magnetic stirring to start the hydrolysis reaction. SiNPs (1 g) dissolved in 25 mL ethanol and sonicated for around 30 min was added to the reaction mix and kept at 70 °C for 23 h under continuous magnetic stirring. The functionalized SiNPs were then dried at 80 °C for 24 h and stored at room temperature. To confirm if the APTES functionalization worked, the samples were tested with the salicylaldehyde test and subjected to FTIR and zeta potential measurements. 

#### 2.1.3. Functionalization of SiNPs with Carboxy (COOH) Groups

The modification of SiNPs with carboxy functional groups was performed by following a slightly modified protocol by Barczak et al. [25]. First, 0.1 g of SiNPs, 0.024 g of Meldrum’s acid (MA), and 33.3 mL of toluene were added to a round-bottom flask attached to a reflux condenser and heated to 110 °C in an oil bath with continuous stirring for 3 h. Then, the yellowish reaction mixture was centrifuged at 6000× *g* and washed once with 1% chloroform (diluted in Millipore water) and twice with absolute ethanol to assure that the unreacted species derived from the Meldrum’s acid decomposition were removed. The washed mixture was then placed into a clean round-bottom flask with 33.3 mL of water and boiled for another 3 h. Finally, the contents of the flask were poured into a crystallization cup and dried overnight at 70 °C. The SiNP_M-functionalized particles obtained were then stored in an airtight container.

#### 2.1.4. Efficiency of Functionalization Reaction

The efficient functionalization of SiNPs with both amino and carboxy groups was confirmed by FTIR, Schiff base reaction and zeta potential measurement. The aqueous NP dispersions were dried overnight at 80 °C, and the dried samples were used for FTIR measurements. The FTIR spectra were recorded using an FTIR spectrometer (Tensor 27, Bruker Optics, Ettlingen, Germany) equipped with an ATR (MIRacle ATR, Pike technologies, Fitchburg, WI, USA) accessory and Opus Software (Bruker Optics Version 6.5, Billerica, MA, USA). The Schiff base reaction was performed with salicylaldehyde to prove the presence of NH_2_ groups with modified concentrations from Cuoq et al. (2012) [26]. Hereby, 10 µg of functionalized particles was added to 1 mL of absolute ethanol and 16 µL salicylaldehyde.

### 2.2. Characterization of SiNP

#### Size, Surface Charge, and Morphology of NPs

The hydrodynamic particle size of the SiNP was determined using nanoparticle tracking analysis (NTA) and dynamic light scattering (DLS). For NTA measurements, the different SiNPs were diluted to a concentration of 10 µg/mL with deionized water and analyzed using the NanoSight LM10 instrument (Malvern, Kassel, Germany) and the NTA 3.2 Dev Build 3.2.16 software (Malvern Panalytical, Malvern, UK). For the measurements, the standard measurement protocol was followed with 5 captures per measurement and 15 s capture duration. The hydrodynamic sizes and zeta potentials of the SiNPs were further confirmed by DLS. A concentration of 100 µg/mL of NPs was prepared in the buffer used for conjugation and the measurements were performed using the ZetaSizer Nano ZS (Malvern Panalytical, Malvern, UK) and the ZetaSizer Software (Malvern, 7.03, Malvern Panalytical, Malvern, UK), modulating the settings for refractive index of the NP composition and dispersant (1.45 for silica).

The primary particle size of the SiNPs was determined by transmission electron microscopy (TEM). For measurement, 2 µL of a 10 µg/mL NP dispersion was dried overnight on a lacy carbon-coated copper TEM grid and imaged using the JEM F200 (JEOL, Freising, Germany) electron microscope in TEM mode operated at 200 kV. Primary particle size was determined by calculating the mean ± SD of minimum 10 particles via image processing with the ImageJ software (NIH, Bethesda, MD, USA) and manual measuring.

### 2.3. Quantification of Endotoxin Contamination

The lipopolysaccharide (LPS) contamination in the synthesized and functionalized particle systems was tested by two different methods in agreement with recommendation for proper endotoxin testing of nanomaterials [27].

#### 2.3.1. Monocyte Activation Test (MAT)

All studies involving human cells were conducted in accordance with the guidelines of the World Medical Association’s Declaration of Helsinki. According to the national regulations, no additional approval by the local ethics committee was required in the case of anonymous blood cells discarded after plasmapheresis (buffy coats). Peripheral blood mononuclear cells (PBMCs) were isolated from blood samples of anonymous donors by density gradient centrifugation. From the PBMCs, monocytes were then purified using magnetically-activated cell sorting by CD14^+^ MicroBeads UltraPure human kit (Miltenyi Biotec, Bergisch-Gladbach, Germany) according to the manufacturer’s protocol. A total concentration of 1.5 × 10^5^ cells/mL was seeded in the wells at a volume of 270 µL per well in cell culture medium containing RPMI, 10% heat-inactivated FCS, 1% 2 mM L-Glutamine, 1% Pen–Strep, and 50 mM 2-mercaptoethanol. The cells were stimulated with 30 µL (final sample concentration 100 µg/mL) of sample and incubated at 37 °C for 24 h. Endotoxin-free water was used as the diluent. After incubation, the supernatants were collected and tested for cytokine (IL-6, TNF-α) release by ELISA (Peprotech, London, UK), since these are the prominent cytokines induced by LPS. An LPS standard curve ranging from 1500 to 0.7 pg/mL on the differently coated cytokine ELISA plates was used to determine the quantitative amount of LPS in the samples.

#### 2.3.2. HEK Blue^TM^ LPS Detection Assay

The HEK Blue^TM^ LPS detection assay, obtained from InvivoGen (San Diego, CA, USA), is a very simple, sensitive, and reliable assay to detect biologically active LPS. Briefly, 20 µL samples were added and a LPS standard curve ranging from 12.5 to 0 ng/mL was made. Endotoxin-free water was used as the diluent. hTLR4 cells were prepared at a concentration of 140,000 cells per mL and 180 µL were then added to the samples, incubated at 37 °C, 5% CO_2_ for 16 h. Afterwards, NF-κB activation was measured in detection medium containing Quanti Blue^TM^ by reading the absorbance at 650 nm. The remaining protocol was followed in accordance with the manufacturer’s instructions.

### 2.4. Binding Efficency

To determine the binding efficiency, 500 µg/mL of NPs were incubated with 100 µg/mL of protein (Bet v 1) in the presence of 0.9% NaCl and 10 mM HEPES buffer pH 7.4 (binding buffer) at a volume of 500 µL at 4 °C for 17 h on a test tube rotator. The recombinant birch pollen allergen Bet v 1 used in this study was prepared as previously described [28]. To determine the total amount of protein bound to the surface of the NPs, the samples were centrifuged twice for 1 h with 16,000× *g* at 4 °C and pellet and supernatant was thoroughly separated. The two-step collection of the supernatant reduces the risk of contaminating the supernatant with the pellet. The bound protein was quantified directly from the pellet using 15% SDS-PAGE by relating it to the concentration of standards using the ImageLab software (version 6.0.1, 2017, Bio-Rad Laboratories, Hercules, CA, USA). In parallel, the unbound protein in the supernatant was quantified using BCA (bicinchoninic acid) assay. From this the percentage of bound protein was estimated indirectly. Hence, testing the amount of proteins left in the supernatant with the BCA assay also allows determine whether the amount of protein in the pellet and supernatant add up to the total amount of Bet v 1 present in the reaction mix during coupling.

### 2.5. Generation of Human Monocyte-Derived Dendritic Cells (moDCs)

PBMCs were isolated from buffy coats by density gradient centrifugation using histopaque-1077 (Sigma, St. Louis, MO, USA). The buffy coats from donors were kindly provided by the SALK (Salzburger Landesklinikum). The monocytes were then separated from the PBMCs using the adherence method. The adherent monocytes were then cultured in dendritic cell medium containing RPMI (Sigma), 10% heat-inactivated FCS (Biowest, Nuaillé, France), 1% 2 mM L-glutamine (Sigma), 1% Pen–Strep (Sigma), 50 mM 2-mercaptoethanol (Sigma) supplemented with 50 ng/mL granulocyte macrophage colony-stimulating factor (GM-CSF) (Life Technologies, Carlsbad, CA, USA) and interleukin 4 (IL-4) (Life Technologies, Carlsbad, CA, USA) for 6 days. The cells were refed with 100 ng/mL of GM-CSF and IL-4 on day 3. The protocol for generation of moDCs is described in detail by Posselt et al. [29].

### 2.6. Viability of Human Monocyte-Derived Dendritic Cells (moDCs) 

The effect of functionalized SiNPs on the cytotoxicity of moDCs was assessed by the commonly used lactate dehydrogenase (LDH) assay, which determines the release of the cytoplasmic enzyme LDH. For analysis, 90 μL of LDH buffer (200 mM NaCl, 80 mM Tris/HCl, pH 7.2) was mixed with 90 μL of the media samples of moDCs stimulated for 24 h. Then, 180 µL of the LDH reaction mixture (0.4 mM NADH and 4 mM sodium pyruvate in LDH buffer) were added and the absorption at 340 nm was immediately recorded and followed over a timeframe of 15 min. Cell viability was calculated by comparing extracellular LDH activities of samples with extracellular LDH activities of moDCs treated with 0.1% Triton X-100 (total LDH release).

Additionally, the viability was determined using the Fixable Viability Dye eFluor506 (eBioscience, Waltham, MA, USA) by flow cytometric analysis using a 510/50 band pass filter. The percentage of viable cells was calculated by gating CD1a on the x-axis, indicating the well-differentiated moDCs and viability stain on the y-axis.

### 2.7. Kinetics and Mechanism of Uptake

To assess the impact of functionalization on the uptake by moDCs, the protein Bet v 1 was fluorescently labelled with pHrodo™ Red, succinimidyl ester (ThermoFisher Scientific, Waltham, MA, USA) according to the manufacturer’s instruction. The detailed protocol for labelling can be found in the supplementary materials file. The labelled protein was bound to SiNPs as described in 2.4. moDCs were seeded in a 24-well plate at a density of 1 × 10^5^ cells per mL, stimulated with 1 µg/mL of Bet v 1 bound to 100 µg/mL of different SiNPs and incubated for different time points (1, 2, 4, 6, 8, 24 h) to assess the kinetics of uptake. The mechanism of uptake was investigated using four different inhibitors, i.e., 2 µM Cytochalasin D (macropinocytosis and phagocytosis) (Sigma); 20 µM chlorpromazine-hydrochloride (clathrin-mediated endocytosis) (Sigma); 1 µM filipin (caveolin-dependent endocytosis) (Sigma); and 10 µM rottlerin (macropinocytosis) (Merck, Darmstadt, Germany) [30,31,32,33]. 

The concentration of inhibitors for the study was determined by testing its effect on the viability of moDCs. The inhibitors were preincubated with the moDCs for the desired time (cytochalasin D for 90 min and chlorpromazine hydrochloride, filipin, and rottlerin for 30 min). The samples were incubated with the cells for a period of 24 h. After the desired incubation time, the amount of pHrodo inside the cells was quantitatively determined by flow cytometry. The gating strategy presented is mean fluorescence intensity (MFI) and this has been obtained by excluding the dead cells, nanoparticles, and doublets. For the inhibition experiments, the Bet v 1 control was considered as 100% uptake control and all other samples were placed in relation to that. The gating strategy is shown in Figure S2 in Supplementary Materials.

### 2.8. Flow Cytometry 

The expression of co-stimulatory molecules was assessed by flow cytometry. moDCs were seeded at a density of 1 × 10^5^ cells per mL in a 24-well plate and incubated with either 1 µg/mL of Bet v 1 bound to 100 µg/mL of SiNP or 100 µg/mL of SiNP without Bet v 1 for 24 h at 37 °C and 5% CO_2_. The cells were then collected and stained for α-HLA-DR APC (Invitrogen, Waltham, MA, USA), Fixable Viability Dye eFluor506 (eBioscience, Waltham, MA, USA), α-CD1a BV421 (Biolegend, San Diego, CA, USA), α-CD86 PE (eBioscience), α-CD40 FITC (Biolegend), and α-CD83 PE-Cy™7 (BD Bioscience, Heidelberg, Germany). The cells were then fixed by adding 4% PFA (Sigma) solution in PBS, before sample acquisition using the FACS Canto II flow cytometer (BD Biosciences). The data thus obtained were analyzed using the FlowJo X 10.0.7r2 software. The data are presented in relation to the controls, which has been obtained by excluding the dead cells, nanoparticles, and doublets. The gating strategy is shown in Figure S2, in addition to the fluorescence minus one (FMO) control in Figure S3.

#### 2.8.1. Cytokine Multiplexing

The 45-Plex Human Procarta-Plex^TM^ (ThermoFisher, Waltham, MA, USA) was used to measure the release of cytokines and chemokines from moDCs activated with SiNPs following manufacturer’s instructions. Shortly, beads were rinsed (PBS, 0.05% Tween-20) and resuspended in the assay buffer (PBS, 0.05% Tween-20, 1% heat-inactivated FCS) (Biowest) before adding 8.34 µL per well into a 96 V-bottom well plate. Afterwards, 15 μL of samples were added, and the plate was incubated on a 500 rpm shaking orbital shaker at 4 °C overnight. The following day, samples were washed three times and resuspended in 15 μL of detection antibody solution before being incubated at room temperature for 30 min. After three further washes, 20 μL of Streptavidin-PE solution (1:1 in assay buffer) was added to each well and incubated at room temperature for 30 min. Lastly, the samples were rinsed three times before being resuspended in drive fluid for testing. The data were processed using Procarta Plex Analyst Software (ThermoFisher, Waltham, MA, USA) and measured on a Luminex Magpix device.

#### 2.8.2. ELISA

ELISA was performed in accordance with the manufacturer’s protocol (Peprotech, London, UK).

### 2.9. Statistical Analysis

Statistical analyses were accomplished with GraphPad Prism 9. For multiple comparisons, one-way ANOVA (Analysis of Variance) followed by a Tukey’s post hoc test was performed; *p*-values ≤ 0.05 were considered as statistically significant (* *p* ≤ 0.05; ** *p* ≤ 0.01; *** *p* ≤ 0.001; **** *p* ≤ 0.0001).

## 3. Results and Discussion

### 3.1. Characterization of SiNPs

**Figure 1:**
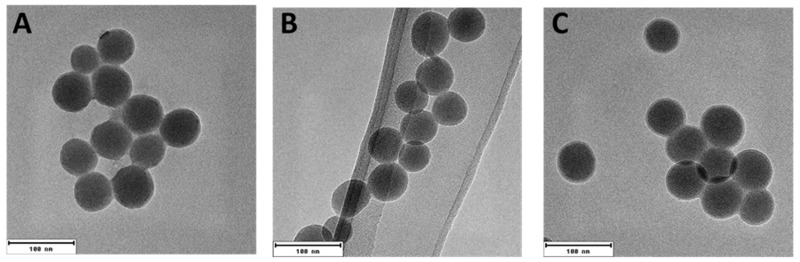
TEM images of the differently functionalized SiNPs for determination of primary particle size (**A**) SiNP, (**B**) SiNP_A, (**C**) SiNP_M, Size bar: 100 nm.

SiNPs synthesized wibyth the Stöber method were chemically functionalized with negatively charged COOH (SiNP_M) and positively charged NH_2_ (SiNP_A) functional groups. As the initial step, the SiNPs and functionalized SiNPs were characterized for their primary size and morphology. The TEM images of the particles displayed a uniform spherical shape with a primary size of 51.02 ± 3.80 nm (SiNP), 47.31 ± 4.72 nm (SiNP_A) and 50.42 ± 4.57 nm (SiNP_M) (Figure 1) [22].

Thereafter, the efficiency of functionalization was tested by FTIR, zeta potential measurements, and the salicylaldehyde test. The FTIR spectra of all the samples exhibited the characteristic peak of silica at 1070 cm^−1^ [34] (Figure S4A–C). SiNP_A showed an additional peak at 1547 cm^−1^ due to NH_2_ bending, which indicated that the functionalization was effective [35] (Figure S4D). For SiNP_M we observed the “C=O” stretch vibration at a wavelength of 1730–1700 cm^−1^; however, the “C–O” stretch and “O–H” bend vibrations at 1320–1310 and 960–900 cm^−1^ were not evident [36] (Figure S4C,D). This might be due to some partial degree of esterification of the carboxyl group when washed with ethanol [37].

To further confirm effective particle functionalization, we determined the pH values of their aqueous suspensions and the surface charge of the particles with the ZetaSizer Nano ZS. SiNP_A exhibited a pH of 8.5 and positive zeta potential (33.4 ± 1.1 mV, whereas SiNP_M exhibited a pH of 4.5 and a negative zeta potential (−26.2 ± 1.8 mV) when compared to the SiNPs that displayed a pH of 7.0 and a negative zeta potential (−25.7 ± 0.5 mV) (Table 1). Moreover, the amino functionalization was tested by a Schiff base reaction. The addition of salicylaldehyde to SiNP_A led to the formation of a bright yellow-colored Schiff base. Centrifugation of the sample resulted in the formation of a yellow-colored pellet and a clear supernatant (Figure S1). This further proved effective amino functionalization.

Finally, the hydrodynamic sizes and distributions of the particles were determined by NTA and DLS (weighted for number and intensity, Figure S5). The SiNPs displayed an intensity-weighted mean hydrodynamic size of 99.0 ± 42.4 nm (SiNPs), 114.5 ± 58.9 nm (SiNP_M), and 108.0 ± 46.4 nm (SiNP_A) by DLS, which were in good agreement with NTA (Table 1). The intensity-weighted values and distributions (Figure S5A–F) show that for most of the particles, small agglomerates were abundant in suspension, whereas the number-weighted distributions (Figure S5G–I) indicate the presence of still non-agglomerated particles, since their sizes match the values obtained from TEM (Figure 1). From all the characterization data, it can be concluded that the functionalization worked efficiently and both the functionalized and SiNPs exhibited a hydrodynamic size of about 100 nm in dispersion.

### 3.2. Lipopolysaccharide/Endotoxin Content in SiNP

**Figure 2:**
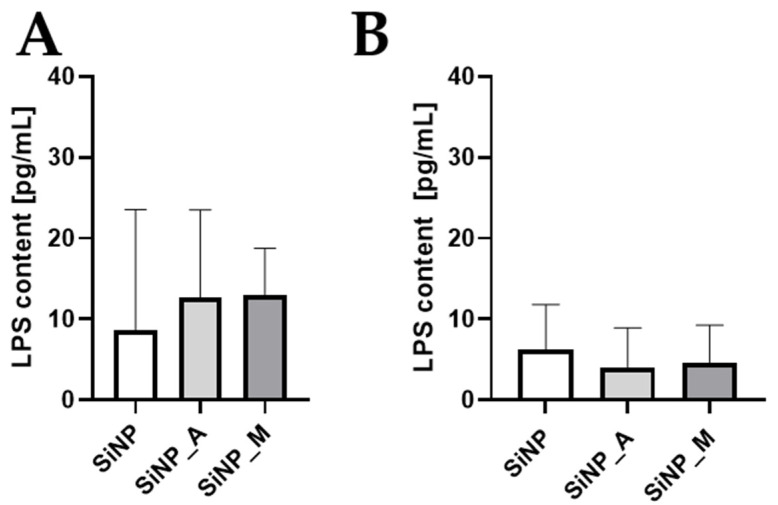
Quantification of LPS content by (**A**) HEK blue assay and (**B**) monocyte activation test (MAT) to analyze LPS contamination in the SiNP samples and Bet v 1. The LPS content was quantified based on LPS standard curves. The data are presented as mean ± SD (*n* = 4).

Lipopolysaccharide (LPS) induces the expression of pro-inflammatory cytokines and surface activation markers at a concentration as low as 20 pg/mL in primary human monocytes [38]. A potential contamination of LPS can cause a health threat in pharmaceutical products or compromise experimental results. Thus, it is necessary to determine the LPS contents in the particle systems, proteins and reagents involved in the in vitro experiments. These were tested by the most sensitive monocyte activation test [39,40] and the HEK Blue^TM^ LPS Detection Kit 2. The results from both the tests indicate that the LPS content in the samples is below 20 pg/mL (Figure 2). Bet v 1 was previously tested after the original preparation and showed LPS values of less than 0.044 pg/µg [41].

### 3.3. Impact of Particle Functionalization on the Binding Efficiency

**Figure 3:**
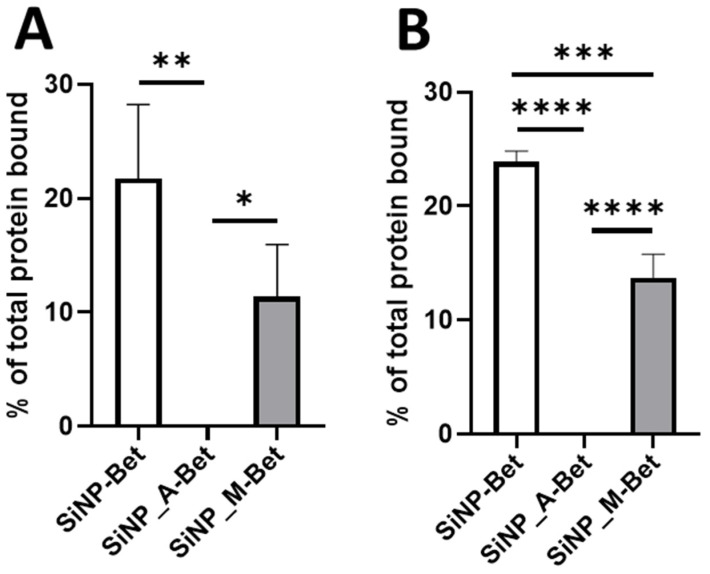
The binding efficiencies of differently functionalized SiNP samples were determined (**A**) directly from the pellet by SDS-PAGE and (**B**) indirectly from the supernatant by BCA assay. The data are presented as mean ± SD (*n* = 4) * *p* ≤ 0.05; ** *p* ≤ 0.01; *** *p* ≤ 0.001; **** *p* ≤ 0.0001.

Nanoparticle surfaces spontaneously adsorb proteins and form a nanoparticle protein corona [42,43]. Allergens are known to form a stable corona on the particle interface [43]. We have previously demonstrated that silica nanoparticles both mesoporous and nonporous adsorb Bet v 1, effectively forming a biocorona [22,44]. The efficiency of binding was determined by SDS-PAGE and BCA assay. From the SDS-PAGE analysis, 21.75 ± 6.50% of Bet v 1 was bound to SiNPs at pH 7.4, whereas the SiNP_M and SiNP_A showed binding efficiencies of 11.41 ± 4.55% and 0.1 ± 0.1%, respectively (Figure 3A and Figure S6). The BCA assay displayed similar yet statistically significant results (Figure 3B). Both SiNP_M and SiNP_A functionalization decreased the binding efficiency when compared to the SiNPs. A similar decrease in serum protein adsorption to COOH-modified mesoporous SiNPs was previously reported by Lin et al. [45] and Beck et al. [46]. While a decreased protein binding was reported to be due to the increasing negative charge on the particles surface, the influence of the particles’ charge on protein binding was not evident in this study. Furthermore, SiNP_A showed almost negligible binding capacity. The pKa of the used SiNP_A is 7.6, based on the components’ ratios during functionalization [47]. Consequently, Bet v 1 displays an isoelectric point (IEP) in the similar range [48]. This would result in hindered binding due to the similarities in their isoelectric points. Here, it is clear that functionalization of SiNPs is altering the binding efficiency.

### 3.4. SiNPs Do Not Affect the Viability of Antigen-Presenting Cells

**Figure 4:**
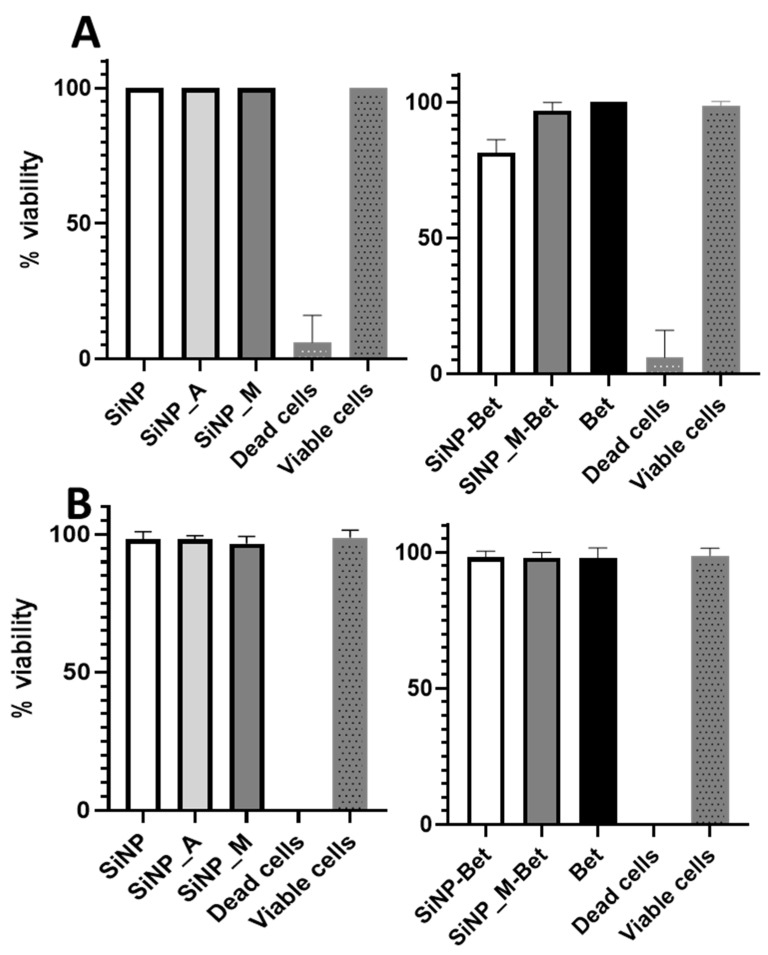
Viability of the moDCs tested with (**A**) LDH assay based on the release of LDH and (**B**) Live/dead staining via flow cytometry after stimulation of moDCs with the samples after 24 h. The negative control in the experiment was dead cells (incubated at 95 °C for 10 min) and the positive control (viable cells) was unstimulated moDCs.

Monocytes were isolated from peripheral blood mononuclear cells (PBMCs) by adherence method and artificially differentiated into immature monocyte-derived dendritic cells (moDCs) by the addition of IL-4 and GM-CSF. The immature moDCs were then stimulated with the samples (100 µg/mL of SiNP, 1 µg/mL of Bet v 1 bound to SiNP, and 1 µg/mL of Bet v 1) for 24 h. The impact of surface functionalization on the viability of moDCs was assessed by LDH assay and flow cytometry after staining the cells with Fixable Viability Dye eFluor506 (live/dead staining). The data from the LDH assay show that the functionalization did not alter the viability of the cells significantly, although we observed a small decrease in viability of moDCs when incubated with SiNP-Bet (Figure 4A). Still, the moDCs proved to be more than 80% viable. The negligible impact of functionalization on the viability was further proved by live/dead staining using flow cytometry analysis (Figure 4B). The gating strategy used for the analysis of the flow cytometry data is represented in Figure S2.

### 3.5. SiNP Adsorption Induces Enhanced Allergen Uptake into Antigen-Presenting Cells Preferentially by Macropinocytosis

**Figure 5:**
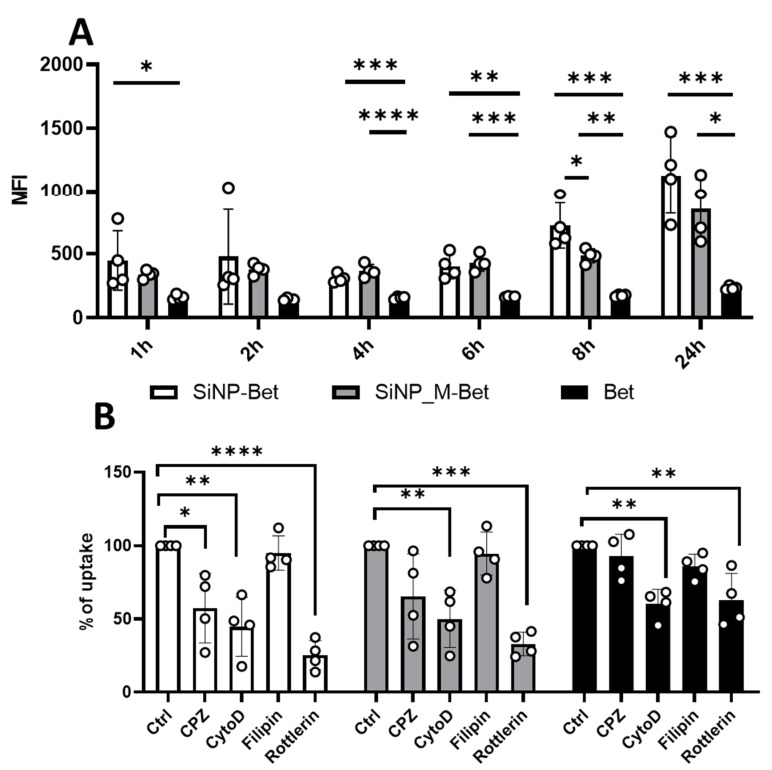
Impact on the uptake of allergen by moDCs. (**A**) Kinetics of uptake represented as mean fluorescence intensity (MFI) over the course of 24 h with SiNP-Bet, SiNP_M-Bet, and Bet. (**B**) Inhibition of uptake with the endocytosis inhibitors chlorpromazine hydrochloride (CPZ), cytochalasin D (CytoD), filipin, and rottlerin after stimulation with moDCs for 24 h (*n* = 4 individual donors). The data are presented as mean ± SD (* *p* ≤ 0.05; ** *p* ≤ 0.01; *** *p* ≤ 0.001; **** *p* ≤ 0.0001).

The first and foremost mechanism contributing to an immune response involves the recognition and internalization of antigens. The internalization of nanoparticles can change based on their physicochemical properties such as size, shape and surface chemistry [6]. We studied the kinetics of uptake using pHrodo-labeled Bet v 1. The labeled Bet v 1 was bound to differently functionalized SiNPs and the samples (Bet v 1, SiNP_M-Bet, and SiNP-Bet) were incubated with moDCs for 1, 2, 4, 6, 8, and 24 h with a concentration of 1 µg/mL of Bet v 1. All particle systems were stable in the suspension for 24 h as controlled by sedimentation analysis employing the silicomolybdic assay (Figure S7) and the protein labeling did not interfere with SiNP adsorption of Bet v 1, as confirmed by SDS-PAGE (Figure S8). Furthermore, the viability of moDCs was not affected when stimulated with the samples (Figure S9). We observed a significant increase in the uptake of allergen when bound to SiNPs at almost all time points (Figure 5A). This increased tendency for uptake was previously reported by Lu et al. (2009) and Heller et al. (2009) where it was shown that uptake is mostly dependent on size, and the maximum uptake by cells occurred when the size of NPs are about 50 nm [21,49]. Similar to Holzapfel et al. (2006), we found an enhanced uptake of protein bound to COOH-functionalized nanoparticles at early time points (up to 8 h) [50]. To clarify further the potential differences in the uptake behavior, we investigated the mechanism of uptake by using specific inhibitors of the major endocytosis mechanism of antigen uptake. The moDCs were pre-incubated with the inhibitors for their required times (Table S1), stimulated with the samples for 24 h and uptake was monitored by flow cytometry. The viability of moDCs remained 90 to 95% when incubated with the inhibitors (Figure S10). Chloropormazine hydrochoride (CPZ) inhibits clathrin-mediated endocytosis, and cytochalasin D (CytoD) inhibits both phagocytosis and macropinocytosis. Filipin inhibits caveolin-dependent endocytosis and rottlerin inhibits macropinocytosis [30,31,32,33]. The results revealed that there was a significant decrease in uptake of SiNP-Bet when incubated with rottlerin (around 75% inhibition), cytochalasin D (around 50% inhibition), and chlorpromazine HCl (40% inhibition). Thus, Bet v 1 molecules adsorbed to SiNPs are taken up mostly by macropinocytosis and Clathrin mediated endocytosis. It has previously been reported by Sahay et al. (2010) [51] that silica-based nanoparticles are internalized with clathrin-mediated endocytosis mechanisms. However, in SiNP_M-Bet we observed inhibition with rottlerin (around 70%) and cytochalasin D (around 50%). Macropinocytosis was thus revealed as the dominant mechanism of uptake for SiNP_M-Bet. Similarly, in Bet, about 40% uptake was inhibited with rottlerin and cytochalasin D, suggesting macropinocytosis as the preferred mechanism of uptake (Figure 5B) [32,52]. Overall, we observed an efficient uptake of Bet v 1 when adsorbed to SiNPs and that macropinocytosis is the preferred endocytosis mechanism for the internalization of Bet v 1. However, we did not observe any significant differences in either the kinetics or mechanisms of uptake between the functionalized SiNP samples, putatively due to negligible differences in size.

### 3.6. SiNP Adsorption and Functionalization Do Not Alter the Maturation of Antigen-Presenting Cells

**Figure 6:**
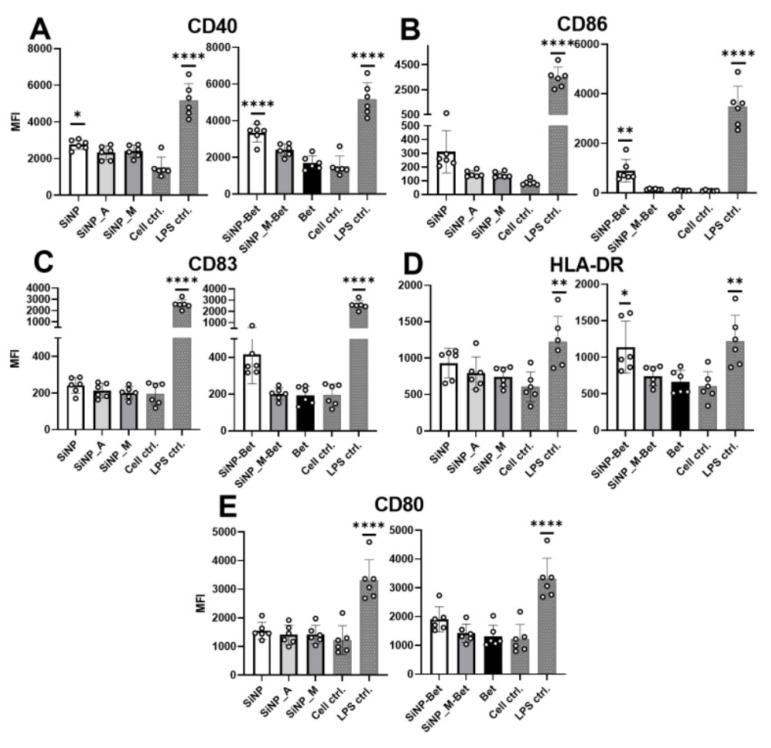
Co-stimulatory molecule expression by stimulated moDCs with functionalized SiNPs and Bet. (**A**) CD40, (**B**) CD86, (**C**) CD83, (**D**) HLA-DR, and (**E**) CD80 were analyzed. LPS was used as the positive control and a statistical significance was observed in comparison to the cell control indicating a successful stimulation. All particles and conjugates were compared to Bet v 1 for statistical analysis (*n* = 6 individual donors) (* *p* ≤ 0.05; ** *p* ≤ 0.01; **** *p* ≤ 0.0001).

**Figure 7:**
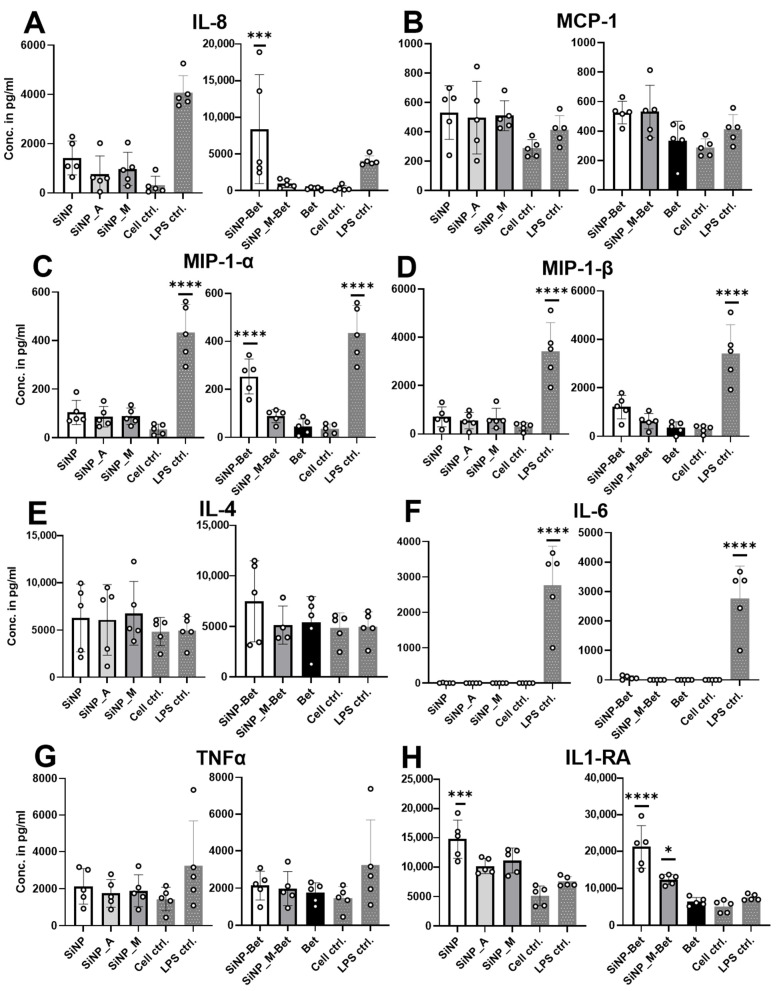
Cyto-/chemokine release of APCs. (**A**) IL8, (**B**) MCP-1, (**C**) MIP-1α, (**D**) MIP-1 β, (**E**) IL-4, (**F**) IL-6, (**G**) TNFα, (**H**) IL1-RA. LPS was used as the positive control and a statistical significance was observed in comparison to the cell control, indicating successful stimulation. The particles were compared to Bet v 1 for statistical analysis (*n* = 5 individual donors) (* *p* ≤ 0.05, *** *p* ≤ 0.001; **** *p* ≤ 0.0001).

In the final part of the study, we investigated the impact of functionalization on the maturation of APCs, which is characterized by the expression of co-stimulatory molecules like HLA-DR, CD86, CD83, and more, along with the release of soluble mediators such as cytokines. These co-stimulatory molecules and cytokines mediate the presentation of antigen to the T cells to modulate an immune response. To study the maturation of APCs, immature moDCs were stimulated with the samples for 24 h and the expression of CD40, CD80, CD83, CD86, and HLA-DR was measured by flow cytometry. The gating strategy is presented in Figure S2. The results obtained clearly showed negligible deviations in the expression of co-stimulatory molecules for Bet v 1 when compared to the untreated moDCs (Figure 6). This is in line with the study of Aglas et al. [53]. Furthermore, as reported before, the positive control (100 ng/mL LPS) showed a significant upregulation of all measured costimulatory molecules [54]. SiNPs showed the tendency to upregulate CD40, CD86, and HLA-DR, particularly when protein was conjugated onto them. In contrast, SiNP_M and SiNP_A caused no upregulation of costimulatory molecules and can thus be considered as a more inert particle system than silica without further chemical functionalization (Figure 6). The release of soluble mediators upon stimulation of moDCs was investigated using the human Procarta Plex Multiplex assay, where 45 different cytokines, chemokines, and growth factors were analyzed; raw data for the multiplex assay are available at https://doi.org/10.5281/zenodo.6473305 (accessed on 20 April 2022). For the control, LPS was used; it induced the release of the cyto-/chemokines and growth factors (IFN-gamma, IL12p70, IL-13, IL-2, IL-4, IL-6, TNF-alpha, IL-18, IL-10, IL-1alpha, IL-1RA, Eetaxin, GRO-alpha, IL-8, IP-10, MCP-1, MIP-1alpha, MIP-1beta, SDF-1alpha, RANTES, HGF, and VEGF-A) in agreement with previous results [55]. When testing the SiNP samples and Bet v 1, we observed the release of the cyto-/chemokines IL-4, IL-6, IL-8, TNF-α, IL-1RA, MCP-1, MIP-1α, and MIP-1β (Figure 7). Notably, we found statistical significance between the Bet v 1-conjugated SiNP samples in IL-8, IL1-RA, and MIP-1α release, suggesting that functionalized particles are immunologically more inert compared to non-functionalized SiNPs when they are used as biopharmaceutical carrier system. To sum up, SiNPs tended to cause the upregulation of costimulatory molecules and increased release of pro-inflammatory cytokines, while these tendencies were not present in the functionalized samples. This indicates that surface functionalization of SiNPs can further decrease the maturation potential of moDCs and can thus be instrumental in preventing the activation and proliferation of T cells, rendering them safer in the context of inducing of an unwanted immune response. It further implies that functionalized SiNPs may remain undetectable as they showed no propensity to activate the innate immune system.

## 4. Conclusions

In this study, we have investigated whether different types of surface functionalization of SiNP impact immune effects by differential activation, i.e., the maturation, of APCs. In addition to first-line safety assessment, early insight on efficacy (second-line) is crucial for decision making in pharmaceutical development; in the context of immunotherapy, this represents the investigation of the immune activating and modulating capacities of administered substances, including nanomaterial platforms. Both the immune activating as well as modulating potentials involve APCs as a crucial player. As a suitable surrogate for human APCs, monocyte-derived dendritic cells were used here. We observed negligible changes induced by the differently functionalized SiNPs in the viability and maturation state of APCs, which is an indication of their immunological inertness. Nevertheless, a significant impact on the binding of proteins to the differently functionalized SiNPs was evident, and could not be simply explained by considering the isoelectric point of protein the net charge of particles upon functionalization. Enhanced uptake was noted for the particle system used here, irrespective of functionalization, while surface functionalization even optimized their immunological inertness, suggesting that SiNPs may be a suitable vehicle for the delivery of biopharmaceutical products, in particular for allergen-specific immunotherapy. Inert (nano)carriers afford the opportunity to intentionally load additional immune modifiers for fine-tuning of desired immunological outcome (e.g., immunomodulation towards a regulatory T helper cell response in allergy or immunosuppression in autoimmunity). As protein binding can be modulated by various types of chemical functionalization, functionalized SiNPs could be useful to deliver a range of biopharmaceutical drugs and lessen the potential immune response. Nevertheless, in vivo studies about the safety and efficacy profiles are required.

## Figures and Tables

**Table 1:** Physicochemical properties of SiNP, SiNP_A and SiNP_M. Size (NTA): Average ± SD of the hydrodynamic diameter mode values of five measurements. Size (DLS): Average ± SD of mean values from intensity- and number-weighted distribution analyses of three measurements. Zeta potential: average ± SD in mV determined when resuspended in buffer (HEPES). PDI: Polydispersity index ranging from 0 (perfectly uniform sample with respect to particle size) to 1 (highly polydisperse sample with multiple particle size populations).

Type	Size [nm] (NTA)	Size [nm] (DLS) Intensity	Size [nm] (DLS) Number	Zeta Potential [mV]	PDI
SiNP	112.2 ± 38.7	99.0 ± 42.4	53.1 ± 0.6	−25.7 ± 0.5	0.34
SiNP_A	206.2 ± 53.5	108.0 ± 46.4	56.3 ± 1.6	33.4 ± 1.1	0.41
SiNP_M	136.8 ± 62.1	114.5 ± 58.9	50.2 ± 7.4	−26.2 ± 1.8	0.38

## Data Availability

All data generated or analyzed during this study are included in this manuscript and its supplementary information files. Raw data of the cyto-/chemokine secretion multiplex assay are available at https://doi.org/10.5281/zenodo.6473305 (accessed on 20 April 2022).

## Supplementary Materials

The following supporting information can be downloaded at: https://www.mdpi.com/article/10.3390/pharmaceutics14051103/s1, Reference [56] is cited in the supplementary materials. Figure S1: Schiff base reaction for testing amino functionalization of SiNP; Figure S2. Gating strategy for moDC experiments; Figure S3. Fluorescence minus one (FMO) controls of moDCs; Figure S4. FTIR spectra of differently functionalized SiNP; Figure S5. NTA and DLS size distribution graphs; Figure S6. SDS-PAGE for estimation of protein binding; Figure S7. Suspension stability test; Table S1. Uptake inhibitors; Figure S8. SDS-PAGE analysis with pHrodo-labelled Bet v 1; Figure S9. Viability of moDCs on exposure to labelled allergen; Figure S10. Viability of moDCs on exposure to selected inhibitors.
