# Supplementary material for: Surface Functionalization of Silica Nanoparticles: Strategies to Optimize the Immune-Activating Profile of Carrier Platforms
Source: Pharmaceutics. 2022 May 21;14(5):1103. doi: 10.3390/pharmaceutics14051103 (PMC9146724; doi:10.3390/pharmaceutics14051103)
Supplement: Supplementary file 1 [file pharmaceutics-14-01103-s001.zip › pharmaceutics-1713885-supplementary.pdf]

Supplementary methods and data

Surface functionalization of silica nanoparticles: strategies to optimize the immune-activating profile of carrier platforms

Benjamin Punz †, Litty Johnson †, Mark Geppert, Hieu-Hoa Dang, Jutta Horejs-Hoeck, Albert Duschl, and Martin Himly

Materials and methods

NP suspension stability analysis / sedimentation assay

NP mixtures were diluted to a final concentration of 100 µg/ml and dispersed in a total of 5 ml moDC cell culture medium. Then 200 µl per sample was taken from the upper section of the suspension and the total silica content was determined by using the silicomolybdic assay as previously reported by *Coradin et al.* and downscaled for microtiter plates[56].

The SiNP containing suspension samples for stability testing were dissolved in 0.5 M NaOH at 95°C for one hour in order to dissolve silica and obtain monosilicic acid. Afterwards the samples were diluted with water at a ratio of 1:15 to obtain 320 µl of diluted samples. These were then incubated for 10 min with 30 µl of a solution containing 0.016 M ammonium molybdate tetrahydrate and 0.7 M hydrochloric acid dissolved in water. Following that, 150 µl of an aqueous solution containing 0.22 M oxalic acid, 0.019 M 4-methylaminophenol sulphate, 0.033 M anhydrous sodium sulphite and 1.8 M concentrated sulphuric acid was added, and the mixture was incubated for 2 h. The absorbance of the samples was measured at λ = 810 nm. Silicon contents were calculated based on known silica standards.

Protein labelling

The allergen was incubated with a 10.2 mM pHrodo™ red succinimidyl ester for 90 minutes at room temperature on a shaker protected from light, the labelled protein was then separated using an illustra NAP-5 column (GE-Healthcare, Zipf, Austria). To elute the labelled protein, 5 mM sodium phosphate buffer (pH 7.4) was used. The protein content was determined using a spectrophotometer (Thermo scientific Nanodrop NO-1000) at 280 nm.The protein-containing fractions were stored at 4°C until further use.

Results

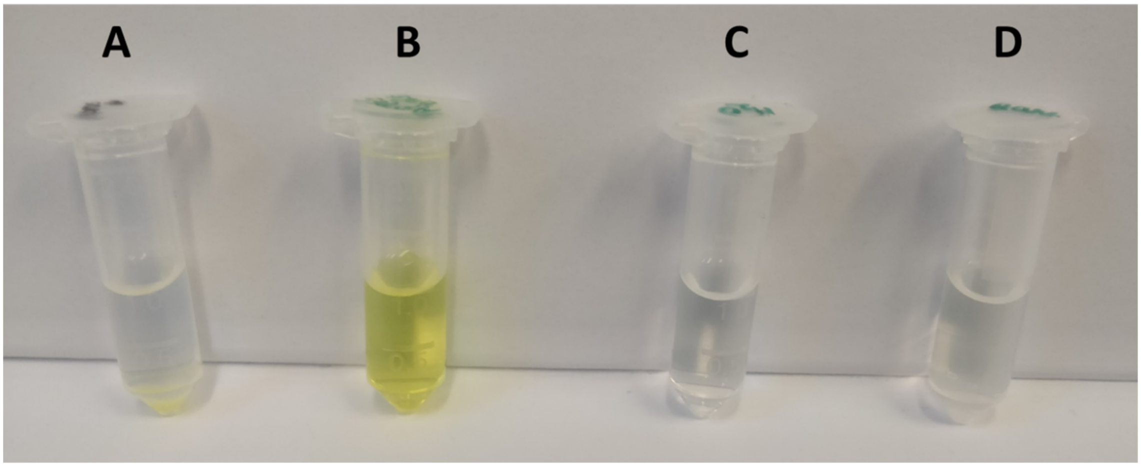

Figure S1. Schiff base reaction for testing amino functionalization of SiNP. (A) SiNP APTES, (B) APTES, (C) Water control, (D) pristine SiNP control indicating a successful functionalization of SiNP_A particles in the yellow pellet.

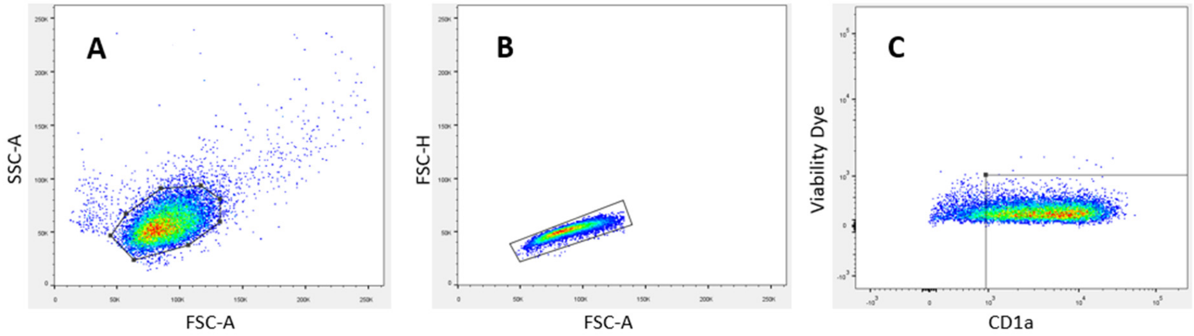

Figure S2. Gating strategy for moDC experiments. (A) FSC-A/SSC-A to remove all cell debris and nanoparticles, (B) FSC-A/FSC-H to remove all doublets, and (C) CD1a/viability dye to remove all dead and non-differentiated cells.

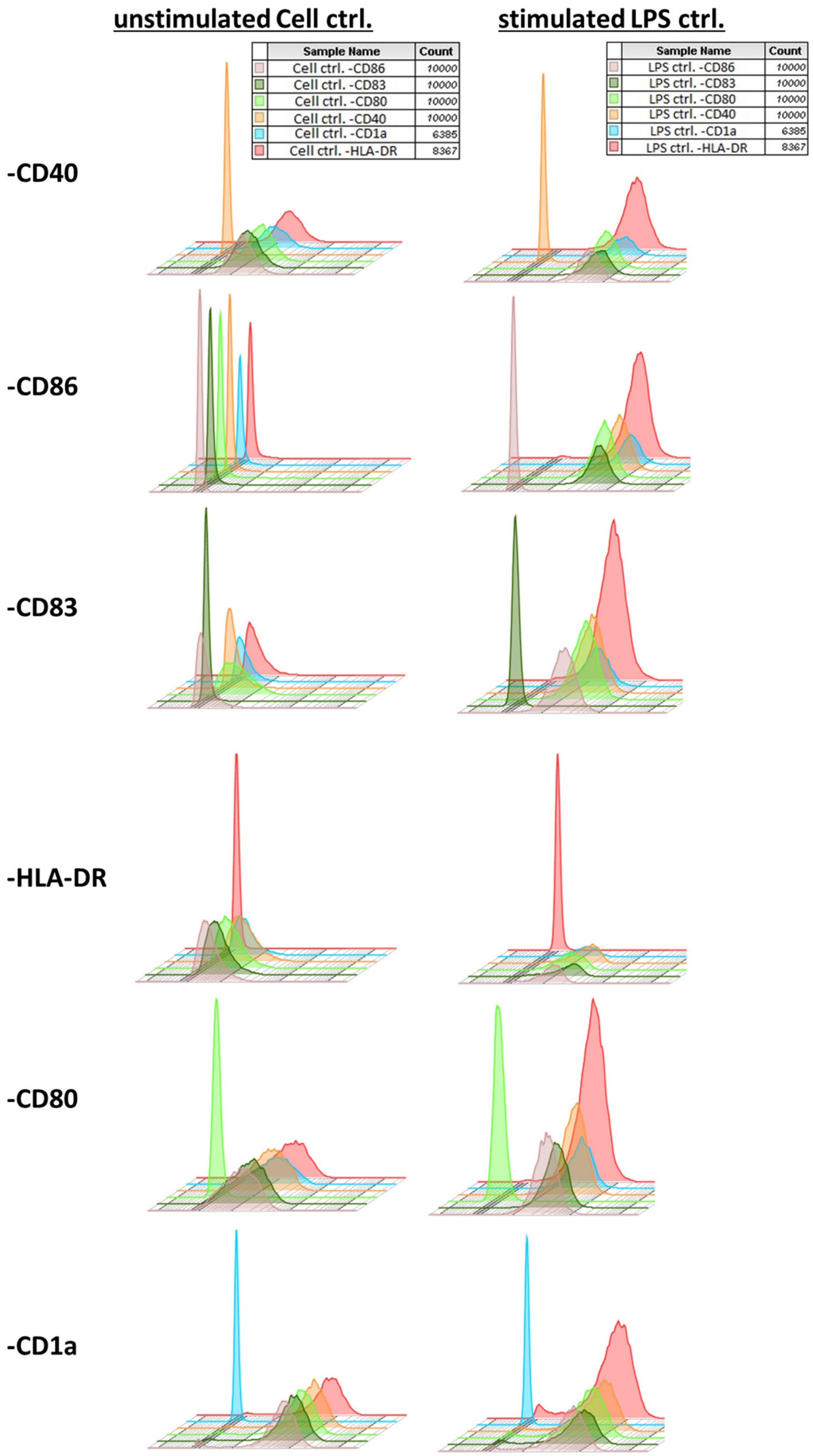

Figure S3. Fluorescence minus one (FMO) controls of unstimulated versus LPS-stimulated moDCs. One dye was removed each from the mix control for potential interference. CD40- FITC-A; CD86- PE-A; CD83- PE-Cy7-A; HLA-DR- APC-A; CD80- APC-Cy7-A; CD1a- BV421-A.

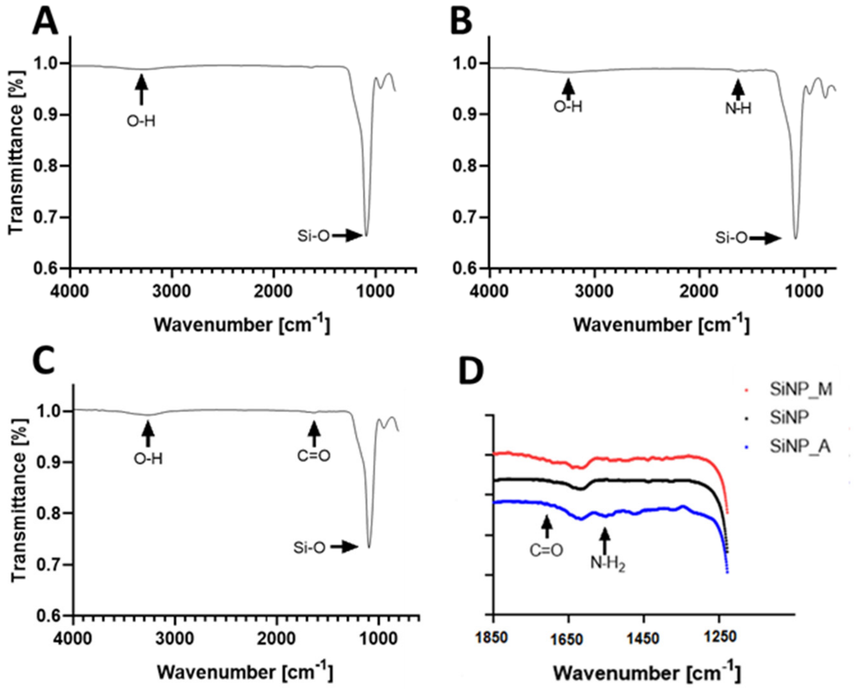

Figure S4. FTIR spectra of differently functionalized SiNP. (A) SiNP, (B) SiNP_A, (C) SiNP_M. Transmittance in percent of the samples plotted against wavenumber in cm^{-1}. (D) showing a close-up on the region ranging from 1850-1250 cm^{-1} directly comparing (A) in black, (B) in blue and (C) in red.

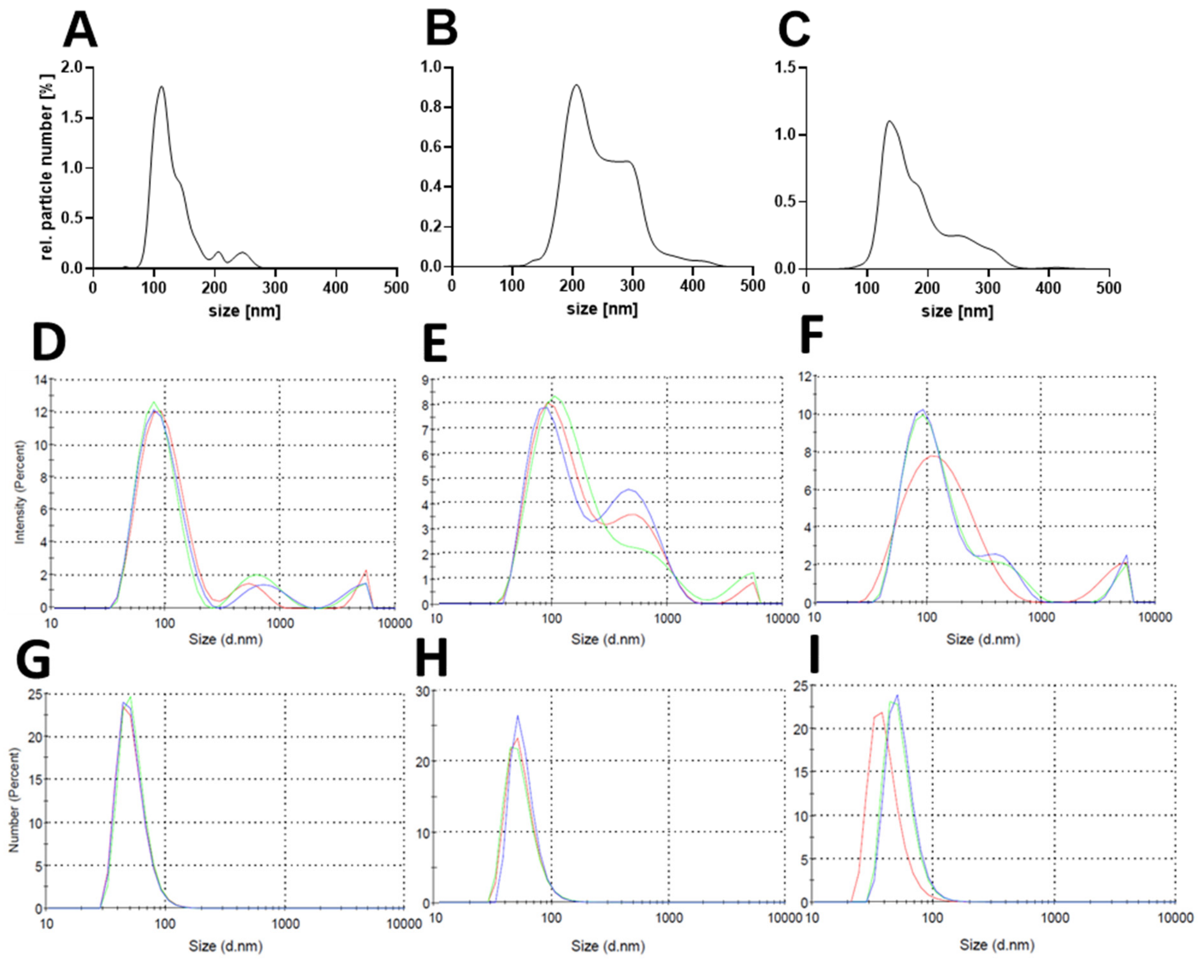

Figure S5. Nanoparticle Tracking Analysis (NTA) and Dynamic Light Scattering (DLS) size distributions from the used particle systems. A,B,C being displayed as the average of the NTA measurements, D,E,F showing the DLS size distribution by intensity (N=3) and, lastly G,H,I showing DLS size distribution by number (N=3). (A,D,G) SiNP, (B,E,H) SiNP_A, (C,F,I) SiNP_M

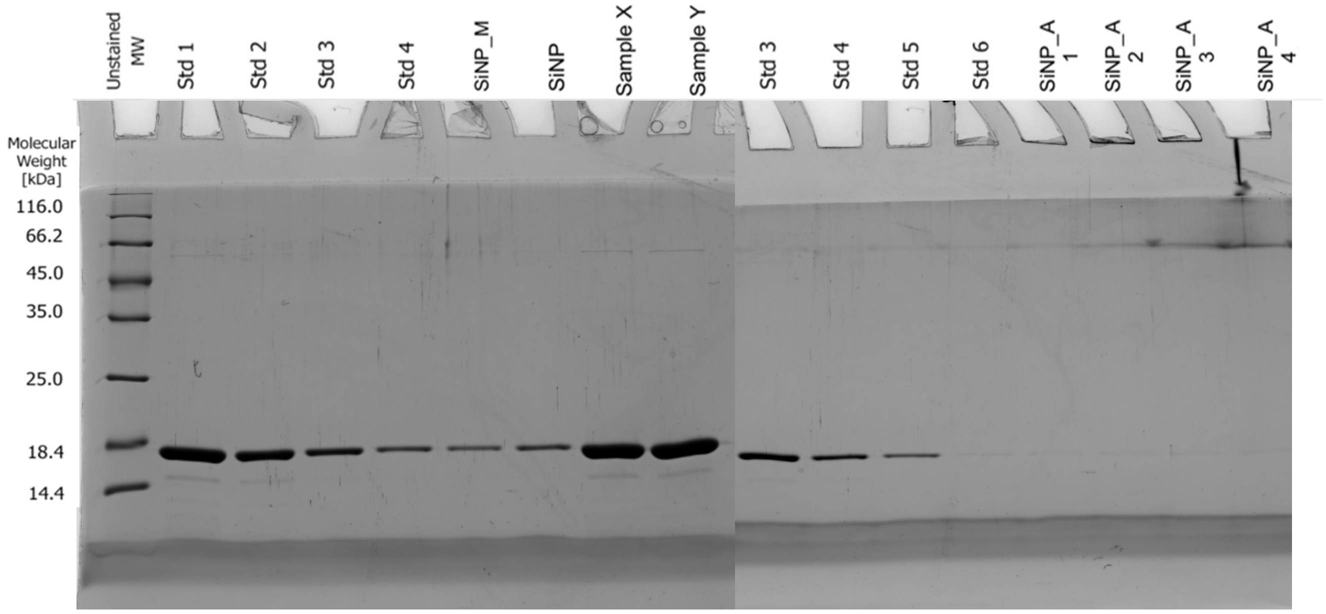

Figure S6. SDS Gels for estimation of bound protein onto particle samples. Standard 1 being the highest concentrated one with a Bet v 1 concentration of 100 µg/mL and further 1:2 dilutions for the consecutive ones. Left to right on the left gel: unstained MW standard, std1 (100 µg/mL), std2 (50 µg/mL), std3 (25 µg/mL), std4 (12,5 µg/mL), SiNP_M, SINP. Right gel: std3 (25 µg/mL), std4 (12.5 µg/mL), std5 (6.25 µg/mL), std6 (3.125 µg/mL), SiNP_A1-4.

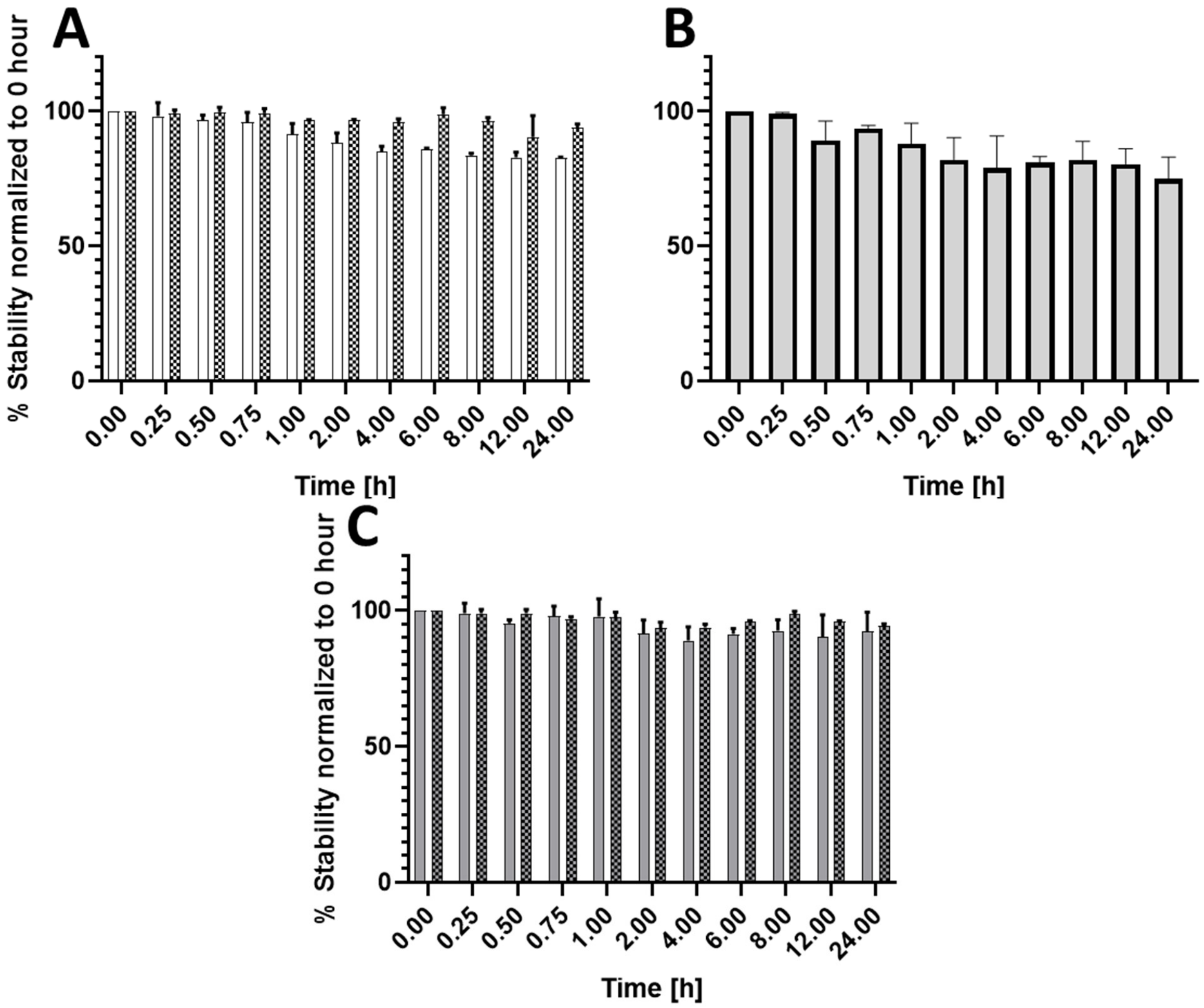

Figure S7. Suspension stability test for 24 h with differently functionalized SiNP. (A) SiNP, (B) SiNP_A, (C) SiNP_M, with (A) and (C), also showing the suspension stability of particles coupled with proteins in the dotted bars (N=3).

Table S1. Uptake inhibitors.

Inhibitor	Mechanism/Inhibition	Pre-incubation time	Concentration (in well)
Cytochalasin D (CytoD)	Macropinocytosis/phagocytosis	90 min	2 µM
Chlorpromazine hydrochloride (CPZ)	Clathrin-mediated endocytosis	30 min	20 µM
Filipin	Caveolin-dependent endocytosis	30 min	1 µM
Rottlerin	Macropinocytosis	30 min	10 µM

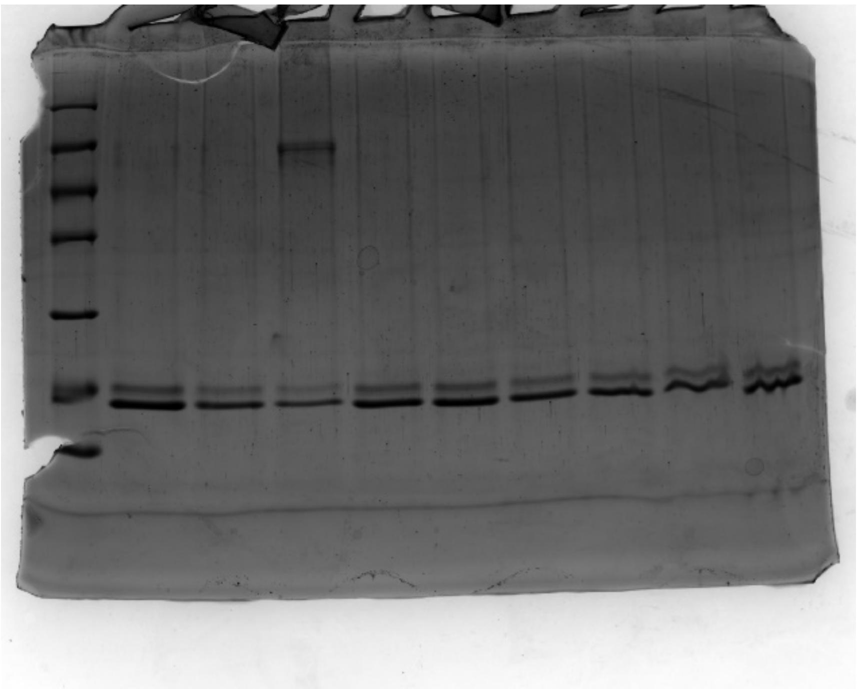

Figure S8. SDS-PAGE analysis with pHrodo-labelled Bet v 1. Left to right: standards 1, 2, 3, SiNP, SiNP, SiNP_M, SiNP_M, extra sample 1 & 2 (redundant for this work).

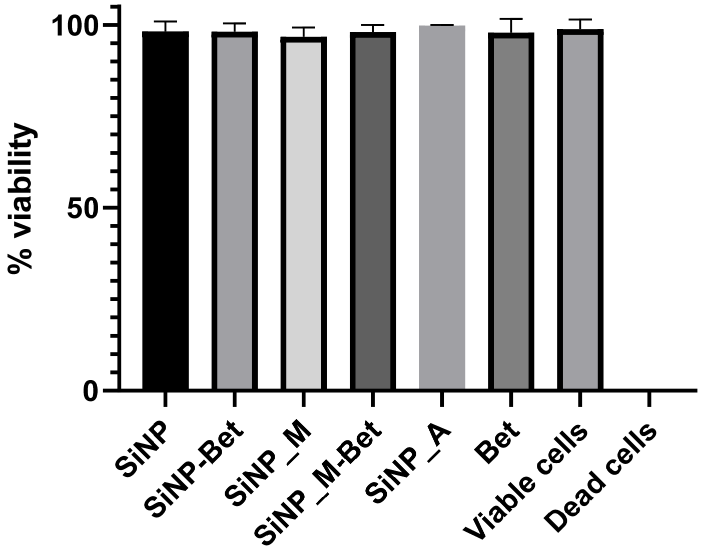

Figure S9. Viability of moDCs upon exposure to labelled allergen coupled to particles tested by life/dead staining *via* flow cytometry.

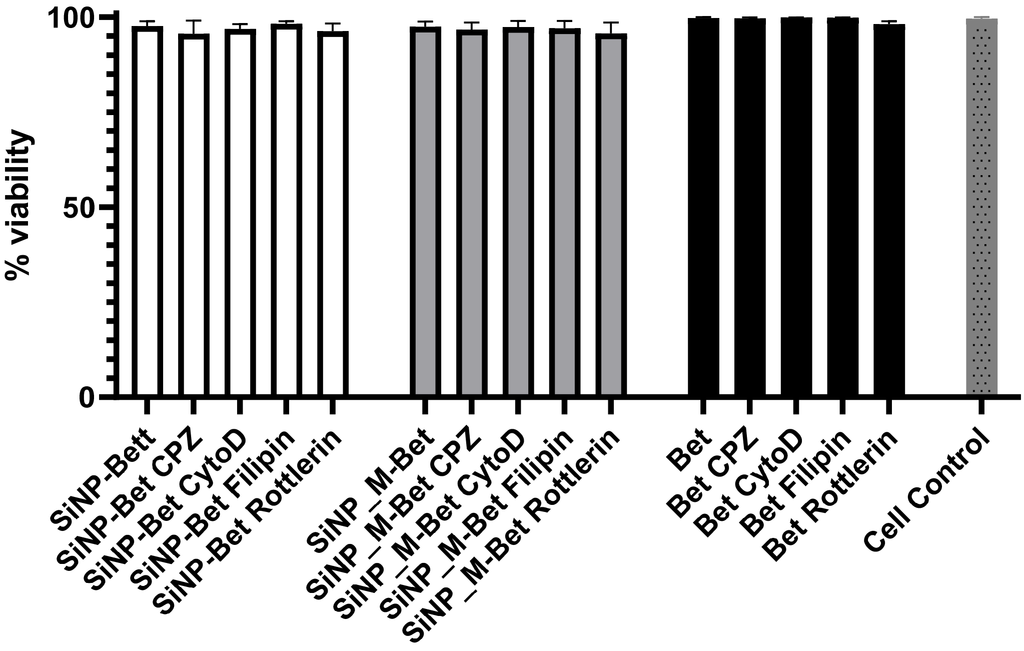

Figure S10. Viability of moDCs after 24 h incubation with selected inhibitors and samples (Cytochalasin D, Chlorpromazine hydrochloride, Filipin, Rottlerin) for the inhibition of selected endocytosis mechanisms (N=4).
